# Testing positive pressure delivered from commercial and WHO-style pediatric bubble CPAP devices

**DOI:** 10.1186/s12887-021-03006-2

**Published:** 2021-11-27

**Authors:** Nicholas A. Ettinger, Nathan Serazin, Richard Nguyen, Jennifer Werdenberg, Minke Huibers, Susan Torrey

**Affiliations:** 1 Division of Critical Care, Department of Pediatrics, Baylor College of Medicine/Texas Children’s Hospital, 6651 Main Street, MC: E1420, Houston, TX 77030 USA; 2 Department of Respiratory Therapy, Texas Children’s Hospital, Houston, TX USA; 3 Division of Hospital Medicine, Department of Pediatrics, Baylor College of Medicine/Texas Children’s Hospital, Houston, TX USA; 4 Senior Global RMNCH Clinical and Technical Advisor, Rice 360° Institute for Global Health, Houston, TX USA; 5 Global Child Health Group, Emma Children’s Hospital, Amsterdam University Medical Center, Amsterdam, the Netherlands; 6 Princess Máxima Center for Pediatric Oncology, Utrecht, the Netherlands; 7 Division of Emergency Medicine, Department of Pediatrics, Baylor College of Medicine/Texas Children’s Hospital, Houston, TX USA

**Keywords:** Pediatrics, Bubble CPAP, Critical care, Global health, Resource limited setting; respiratory failure

## Abstract

**Background/aim:**

Low-cost commercial bCPAP devices have been deployed in resource-limited settings to treat neonatal respiratory failure. The use of these devices has increased access to pediatric respiratory support for infants. However, constrained resources may result in substitution of recommended consumables and/or use in older age groups. We hypothesized that commercially available bCPAP devices, the standard WHO-style device and various improvised adaptations would all generate effective, safe positive pressure at the patient interface.

**Methods:**

Performance of 2 commercially available bCPAP devices was tested against the standard WHO-style bCPAP device, as well as several improvised modifications of these devices, by measuring positive pressure delivered at the patient interface. Variables tested included different flow rates, patient interfaces and respiratory circuit tubing.

**Results:**

Both commercial devices utilized according to manufacturer recommendations generated the expected positive pressure at the patient interface. When testing the recommended WHO-style bCPAP device with recommended materials as well as other improvised modifications, we found variable and potentially unpredictable generation of positive pressure at the patient interface.

**Conclusions:**

Modified or improvised bCPAP devices should be used with extreme caution as the support provided may be more or less than expected depending on respiratory tubing and flow rates employed. Our data support the effectiveness of bCPAP in newborns and young infants. But, to our knowledge, there are no bCPAP patient interfaces for older children effective with low liter flow devices. Therefore, based on these results, we recommend against using WHO-style bCPAP devices for non-infant patients with respiratory failure and instead recommend using standard oxygen therapy with nasal cannulae or face-masks, as well as early consideration of transfer to a higher level of care.

## Background

Respiratory diseases are the most common cause of morbidity and mortality in children under 5 worldwide [1]. In resource limited settings (RLS), options for respiratory support are often constrained. Many locations lack both expertise and access to invasive respiratory support and/or basic pediatric intensive care [2]. Bubble Continuous Positive Airway Pressure (bCPAP) has been proposed as an alternative to meet the need for respiratory support for infants and small children for which several commercial, durable, low-cost devices are available (e.g., Pumani) [3–6]. Studies have demonstrated that bCPAP can be effective, safe and can reduce morbidity/mortality in infant populations [3]. However evidence is mixed regarding the efficacy of bCPAP to improve outcomes in older children with respiratory failure in RLS [7, 8].

In addition to commercially available devices, instructions on how to create “low-tech” modified bCPAP devices with readily available resources have been published, [9] endorsed by the WHO (Figs. 16 & 17 [10]) and promulgated in pediatric global health training websites [11]. In practice, these devices may be subject to large variability in construction based on the availability of specific equipment. This may variably affect the amount of actual positive pressure delivered. Oxygen source, delivered liter flow rates (liters per minute, lpm), circuit tubing, nasal or facial interface, miscellaneous connections, and the reliability of the bubble CPAP chamber are several of the patient-independent variables subject to potential local modification. The efficacy of positive pressure generation at the patient interface by variations of WHO-style devices has been incompletely studied [12]. The aim of our study was to measure the amount of positive pressure generated by commercially available bCPAP devices, the standard WHO-style device and various improvised adaptations. We hypothesized that all would generate effective positive pressure (PP) matching the submersion depth at the patient interface.

## Materials and methods

### Control devices

To test our hypothesis, we used the Fisher & Paykel (F&P) Healthcare Bubble CPAP with Flexitrunk™ and Pumani Bubble CPAP devices as controls. The F&P device was chosen as a representative device used in high-resource settings. It requires a standard high-flow/high-pressure air/oxygen wall interface for air flow along with a proprietary humidifier chamber and self-contained pressure release valve (valve closed for all F&P device testing). The Pumani bCPAP apparatus is a low-cost, durable, Conformitè Europëenne-approved commercial apparatus specifically designed for RLS [4, 5]. It has a self-contained electric flow pump for air flow. Each was assembled with the manufacturer provided components (respiratory tubing, connections, humidifiers, patient interfaces, etc.) according to the manufacturer’s specifications. Both were tested at air-flows recommended by the manufacturers (8 lpm for the F&P device and 6–8 lpm for the Pumani).

### Patient interfaces tested for the control devices (Table 1, Fig. 1)

For the F&P device, we tested F&P nasal prongs (sizes 3020, 3520, 4030, 4520, 5050 and 6570, Fig. 2) and F&P nasal masks (sizes small, medium, large, extra-large, Fig. 3). For the Pumani device, we tested two of the standard Pumani nasal prongs (Hudson, sizes 0[small], 5[large], Fig. 4).

**Table 1:** Configurations and Variables Tested – commercially available devices and improvised modifications

Variation	Air Flow Source	bCPAP Chamber	Tested Device Tubing	Patient Interfaces Tested
**Commercially Available Devices**				
**Fisher & Paykel (F&P)**	Standard high-flow/high pressure air/oxygen wall interface	F&P bubble CPAP chamber (F&P# BC100)	F&P bCPAP tubing	• F&P Nasal Prongs • F&P Nasal Masks • Standard Resuscitation Mask
**Pumani**	Internal air flow pump +/− supplemental low-flow O_2_	Pumani bubble CPAP chamber	• Standard Pumani tubing • Standard Pumani tubing + F&P Flexitrunk™ connector	• Pumani Nasal Prongs • F&P Nasal Masks • Sleepweaver™ BIPAP mask
**WHO-Style Devices**				
**WHO**	Standard low-flow wall oxygen flow-meter (1–10 lpm)	F&P bubble CPAP chamber	Pediatric & Adult size CareFusion AirLife™ nasal cannulae	Cut nasal cannulae
**Improvised WHO**	Standard low-flow wall oxygen flow-meter (1–10 lpm)	F&P bubble CPAP chamber	F&P bCPAP tubing	High flow nasal cannula (West Med)
	Standard low-flow wall oxygen flow-meter (1–10 lpm)	F&P bubble CPAP chamber	Pumani tubing	Pumani Nasal Prongs

**Fig. 1:**
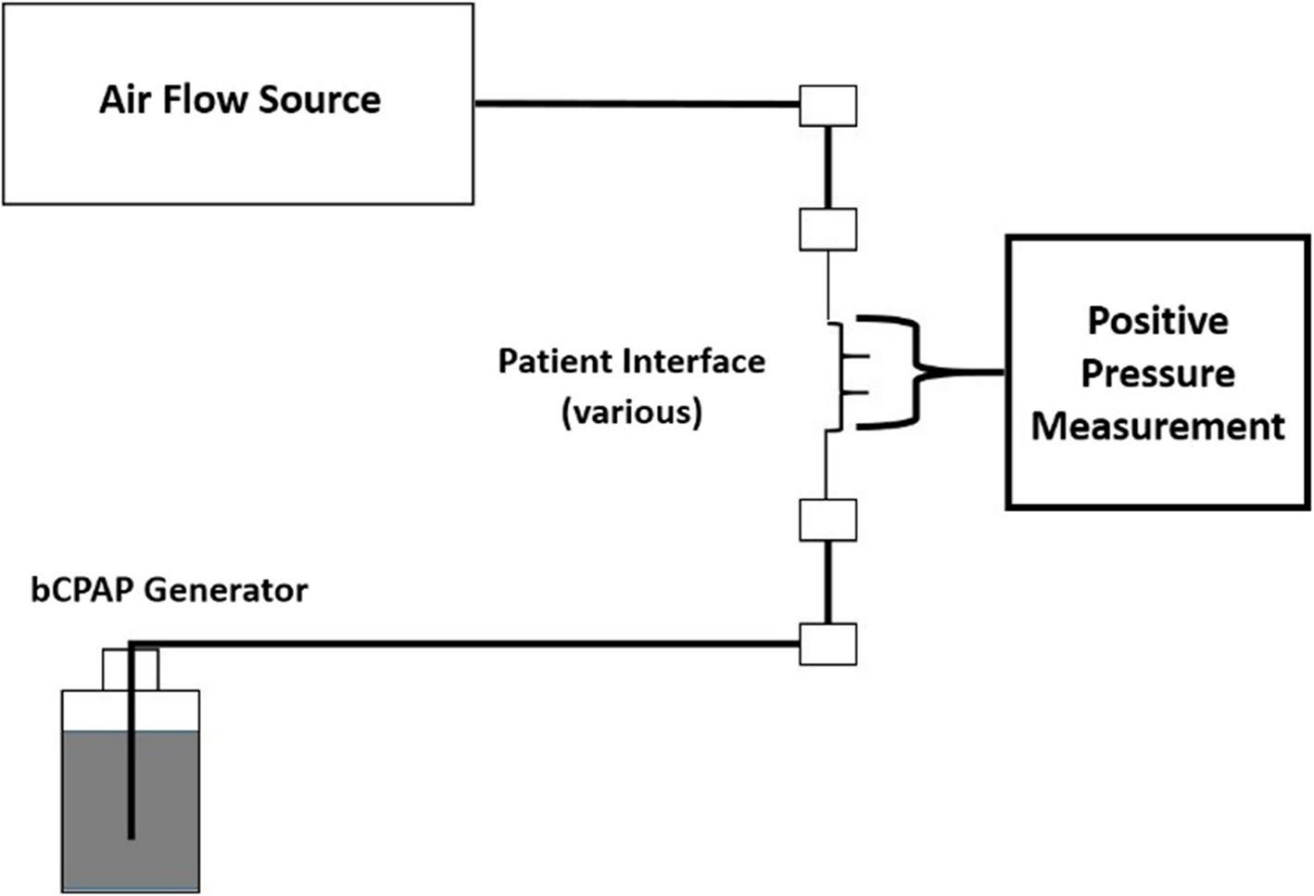
Generalized schema for all tested variations. Squares represent join points between device components – air flow source, bCPAP chamber, inspiratory/expiratory limb tubing and patient interface

**Fig. 2:**
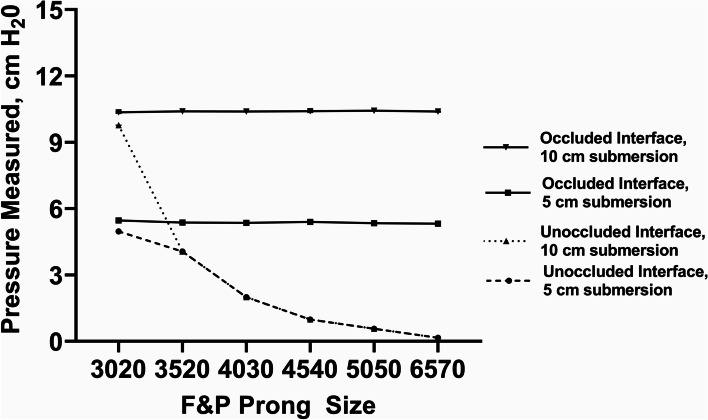
F&P bCPAP device with a F&P circuit connected to F&P Nasal Prongs - Measured Pressure [mean (SD), cm H_2_0]. Testing performed at 8 lpm with two submersion depths – 5 cm H_2_0 and 10 cm H_2_0. X-axis is categorical and not scaled to prong dimensions

**Fig. 3:**
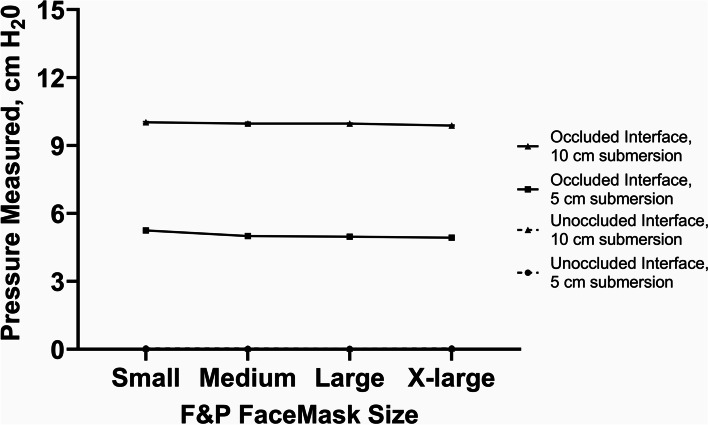
F&P bCPAP with a F&P circuit connected to F&P Nasal Masks - Measured Pressure [mean (SD), cm H_2_0]. Testing performed at 8 lpm with two submersion depths – 5 cm H_2_0 and 10 cm H_2_0

**Fig. 4:**
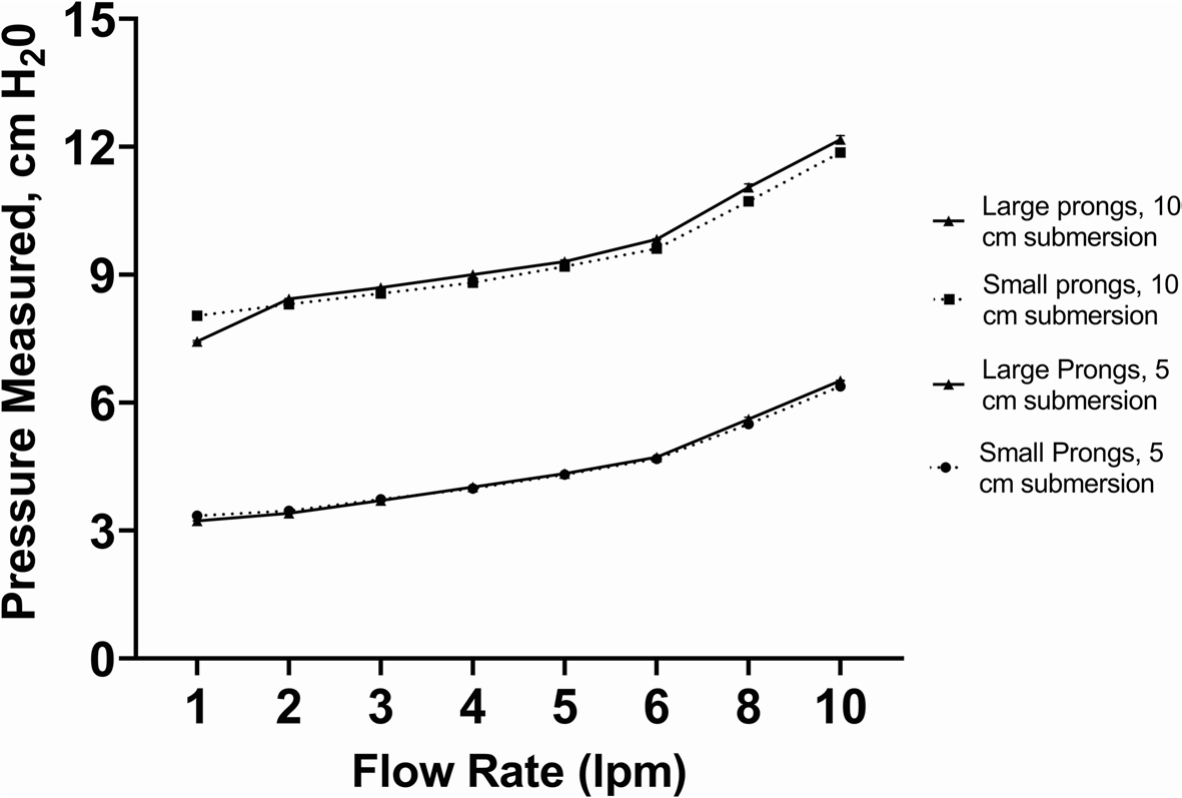
Pumani bCPAP device with a Pumani circuit connected to small and large Pumani Nasal Prongs - Measured Pressure [mean (SD), cm H_2_0]. Testing performed at two submersion depths – 5 cm H_2_0 and 10 cm H_2_0

### Test devices

The test devices (Table 1, Fig. 1) included the (#1) WHO-style recommended bCPAP device [9] and (#2) improvised modifications to the WHO-style device, (#3) the F&P device with modifications and (#4) the Pumani device with modifications. Our choice of improvised modifications was informed by observations in RLS at the bedside by our author group.

**Fig. 5:**
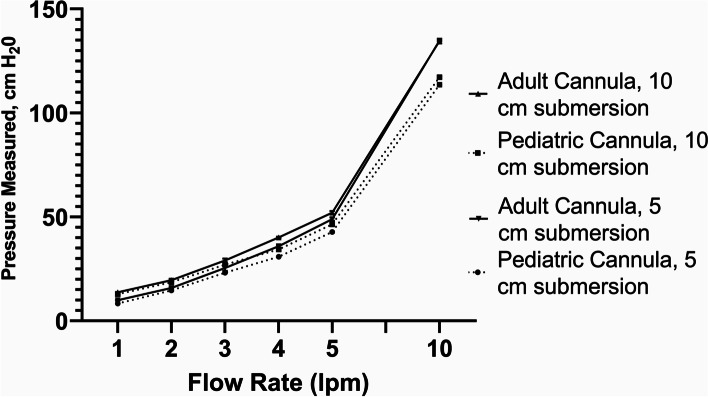
WHO-style bCPAP device – Measured Pressure [mean (SD), cm H_2_0; note difference in scale]. Testing performed at two submersion depths – 5 cm H_2_0 and 10 cm H_2_0

For the WHO-style recommended bCPAP device (#1), we followed instructions as outlined in several commonly referenced global health sources, [9, 10] using a standard low flow wall oxygen source (1–10 lpm) with standard adult and pediatric size nasal cannulae (CareFusion Air*Life*™). The distal cut end of the nasal cannulae was submerged to a depth of 5 or 10 cm in the F&P bubble CPAP chamber for testing (Fig. 5).

**Fig. 6:**
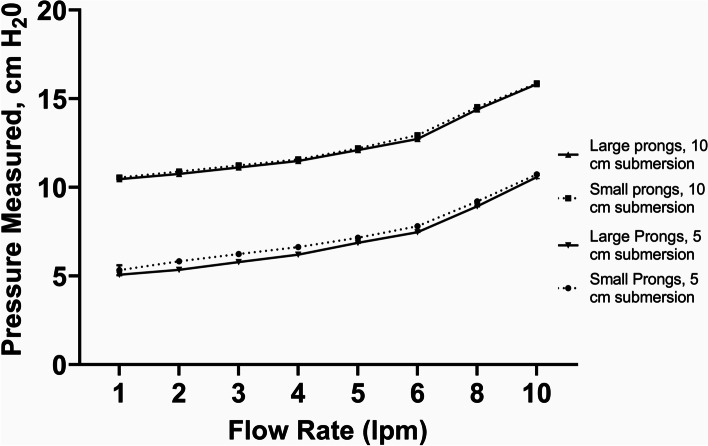
WHO-style bCPAP device with a Pumani circuit connected to small and large Pumani Nasal Prongs - Measured Pressure [mean (SD), cm H_2_0]. Testing performed at two submersion depths – 5 cm H_2_0 and 10 cm H_2_0

**Fig. 7:**
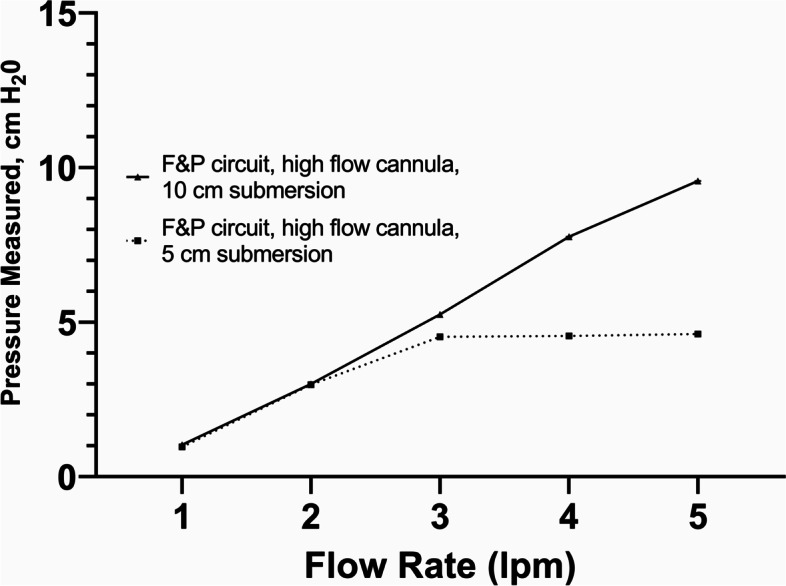
WHO-style bCPAP device with an F&P circuit connected to a standard high-flow nasal cannula - Measured Pressure [mean (SD), cm H_2_0; note testing only performed at flow rates < 5 lpm]. Testing performed at two submersion depths – 5 cm H_2_0 and 10 cm H_2_0

For improvised modifications to the WHO-style device (#2), we tested the following modifications to the inspiratory and expiratory limbs as well as to the patient interface: (#2a) substituting the Pumani respiratory circuit (tubing with a larger diameter than that recommended in the WHO-style device) connected to Pumani small and large nasal prongs in lieu of a standard low-flow nasal cannula (Fig. 6) and (#2b) substituting the F&P respiratory circuit (tubing with a larger diameter than that recommended in the WHO-style device) connected to a standard pediatric high flow nasal cannula (WestMed) in lieu of a standard low-flow nasal cannula over a range of flow rates (Fig. 7).

**Fig. 8:**
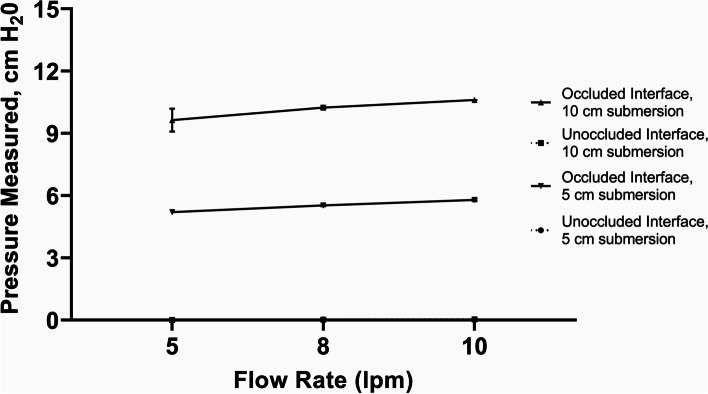
F&P bCPAP device with F&P circuit connected to a standard Ambu-bag resuscitation face mask - Measured Pressure [mean (SD), cm H_2_0]. Testing performed at two submersion depths – 5 cm H_2_0 and 10 cm H_2_0

For the F&P device with modifications (#3), we tested the F&P device substituting a standard Ambu-bag resuscitation mask attached to the end of the F&P respiratory circuit in lieu of the F&P nasal masks (Fig. 8).

**Fig. 9:**
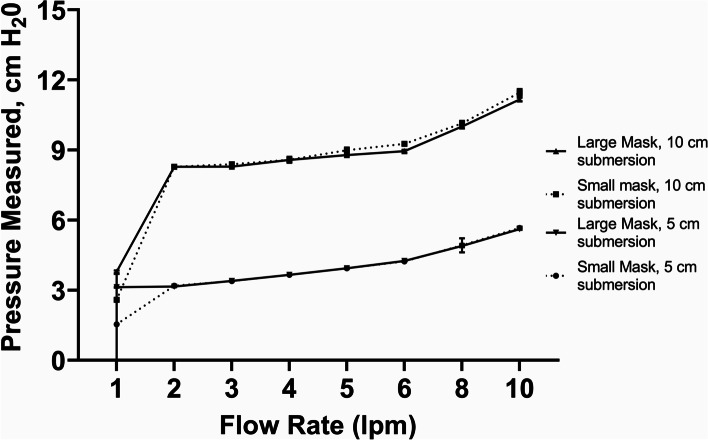
Pumani bCPAP device with a Pumani circuit connected to small and large F&P Nasal Masks - Measured Pressure [mean (SD), cm H_2_0]. Testing performed at two submersion depths – 5 cm H_2_0 and 10 cm H_2_0

For the Pumani device with modifications (#4), we tested the Pumani device with Pumani circuit connected to F&P nasal masks via an F&P connector in lieu of Pumani nasal prongs (Fig. 9). We also tested the Pumani device using Pumani tubing connected to a typical commercially available CPAP/BIPAP mask used in older children (SleepWeaver®, Circadiance, Export, PA, data not shown).

### Testing process

The performance of all bCPAP devices was tested by measuring PP delivered at the patient interface using an American Healthcare Products Calibration Analyzer (Timeter Instrument Corporation; series RT-200). Patient interfaces were connected directly to the measurement inlet probe of the analyzer without leakage to simulate 100% seal of the patient interface to the patient. Although we did not measure this formally, all device variations, tubing and patient interfaces were examined to ensure that the connections were tight and there was no obvious leakage. For the variations tested with the F&P device, we were able to test positive pressure delivered under conditions where the prongs or masks were “fully occluded” vs. “fully unoccluded” to examine the range of possible PP generated as there are built-in access ports as part of the F&P tubing. To test “fully occluded” scenarios we gently occluded the nasal prongs with our fingers. We occluded the nasal masks as well as resuscitation mask by pressing them gently on a flat smooth surface to ensure 100% seal. We acknowledge that this in vitro testing model is static testing and poorly replicates true bCPAP interactions with a live patient. However, the aim of our study was to measure the PP generated by several variations of bCPAP devices that our author group has observed used in real clinical practice in RLS.

### Flow rates

Anecdotally, our author group has seen providers in RLS turn flow rates up intending to provide more support to patients. Typical oxygen sources in RLS include oxygen cylinders or oxygen concentrators. Cylinders deplete quickly if used with flow rates > 10 lpm and oxygen concentrators most commonly generate < 10 lpm. Therefore, to simulate this observation, we varied the flow rates between 1 and 10 lpm.

### Submersion depth

Testing for all variations was performed at two submersion depths – 5 cm H_2_0 and 10 cm H_2_0 in the bubble CPAP chamber (F&P or Pumani, see Table 1).

### Analysis

Each condition was tested three times and the PP delivered was recorded. The analyzer was zeroed to room air between each measurement. Mean and standard deviation data were graphed using Prism v9.0 (GraphPad Software).

## Results

To test our hypothesis, we compared PP delivered at the patient interface by the two commercially available bCPAP devices (Table 1, Figs. 2, 3 and 4) to PP delivered at the patient interface from the WHO-style, “low-tech” device and several improvised variations (Table 1, Figs. 5, 6, 7, 8 and 9).

### Control devices

Testing the F&P device as recommend by the manufacturer with a range of F&P nasal CPAP prongs or with a range of F&P nasal masks, the delivered pressure matched the pressure set by the submersion depth in the bubble CPAP chamber and there was active, visible bubbling in the CPAP chamber (Figs. 2 and 3; 100% occlusion; recommended flow rate of 8 lpm flow). Of note, with larger nasal CPAP prong sizes and with all the nasal mask sizes, there was only visible bubbling when the prongs or masks were occluded. However, with the smallest F&P nasal prongs (size 3020) there was visible bubbling in the CPAP chamber even with no occlusion of the prongs (Fig. 2).

For the Pumani device, we observed that with 100% occlusion of the prongs, there was bubbling in the chamber and measured pressures approximated or slightly exceeded (at higher flow rates) the submersion depth (Figs. 4 and 5 cm and 10 cm H_2_0). With the prongs unoccluded, there was no bubbling at all tested flow rates. There was no difference in measured pressures between the smallest and largest Pumani nasal prongs (sizes 0[small], 5[large]) at either submersion depth or over a range of flow rates (1–10 lpm, manufacturer’s recommend flow rate is 6–8 lpm).

### Test devices

For the WHO-style bCPAP devices (#1), the measured pressures at the end of the nasal cannulae rapidly exceeded the submersion depths in the F&P bubble CPAP-chamber even at low flow rates (Fig. 5) using both pediatric and adult nasal cannulae attached to a standard low-flow wall oxygen source at 1–10 lpm. For the improvised WHO-style device using a low flow wall-oxygen source with the Pumani respiratory circuit and Pumani nasal prongs (#2a), the measured pressures approximated the submersion depths at lower flow rates, but began to exceed the submersion depths at higher flow rates (Fig. 6) for both small and large nasal prongs. For the variation utilizing F&P tubing connected to a standard high flow nasal cannula (#2b), the measured PP was less than the set submersion depths at flow rates < 5 lpm (Fig. 7).

For the improvised variation of the F&P device with the F&P circuit connected to an Ambu-bag resuscitation mask as the patient interface (#3), the measured pressure matched the set depth in the CPAP chamber over a range of flow rates (5–10 lpm), if there was 100% occlusion of the face mask (Fig. 8).

For the improvised variation of the Pumani device (#4), using F&P nasal masks as the patient interface, the measured pressures approximated the submersion depth in the Pumani bubble-chamber at all flow rates except at 1 lpm (Fig. 9). The Pumani device combined with SleepWeaver® nasal mask was unable to generate any measurable PP at any flow rate (data not shown).

## Discussion

Evidence to support the use of bCPAP for older infants and children in RLS is limited, has variable results and is of lower quality [7, 8]. Based on our informal observations of the use of various WHO-style bCPAP devices used outside of the neonatal age range, we were concerned that the support provided for those older patients (i) may not achieve any PP benefiting the patient and thus may be wasting oxygen supply resources or (ii) may delay transfer to a higher level of care during a trial of therapy or (iii) may potentially be providing excessive PP which may be harmful to the patient.

Our results demonstrate that for the commercial bCPAP devices using flow rates recommended by the manufacturers, both F&P and Pumani devices delivered at least the expected PP at the patient interface matching the submerged depth in the bubble-chamber, although the Pumani device had a steeper increase in resulting pressures (Figs. 2, 3 and 4). The etiology behind this performance difference is unclear although could be related to the performance of the flow pump inside the Pumani device at higher flow rates or the higher expiratory tubing resistance in the Pumani device [13]. It is important to note that with the F&P device using the smallest size nasal prongs (size 3020), we observed bubbling in the bubble-chamber without occlusion, likely secondary to the intrinsic high resistance of this prong size [14]. The intrinsic resistance creates the potential for a provider to misjudge the level of PP support actually being provided to the child, regardless of the actual leakage at the patient interface [15]. Chamber bubbling was a reliable proxy for occlusion with the Pumani device, with the F&P nasal masks and with the resuscitation face mask (Figs. 3, 4 and 8). However, we strongly recommend against using the resuscitation face mask in this manner because of significant safety concerns related to possible hypercarbia, risk for aspiration or gastrointestinal perforations [7, 16].

With the standard WHO-style bCPAP device, the cut nasal cannulae generated PP that far exceeded the set depth in the F&P bCPAP chamber (Fig. 5). The data from the pediatric cannula were uniformly lower than the data from the adult cannula, suggesting the possibility of unmeasured leakage. These high measured pressures were also observed in a recent publication comparing narrow and wide-diameter bCPAP circuits [12]. The high PP occurred only with 100% occlusion of the nasal cannulae, but these data raise concern that in small infants, especially with higher flow rates, the pressure delivered could create discomfort, intolerance or barotrauma in an infant leading to use of resources without benefit. Testing a variation of the standard WHO-style bCPAP device with larger diameter F&P tubing (a combination observed in RLS by our group) prevented the generation of excessive PP values (Fig. 7). However, this improvised device was not able to generate appropriate pressures at lower flow rates, likely secondary to leaks in the system, although we did not formally test for this possibility. However, this was still a concerning result as some providers may think that a low flow rate could be a beneficial strategy to stretch limited oxygen resources while still providing PP. Our results demonstrate that using low flow rates for this variation provides oxygen only with little to no PP benefit. An improvised variation employing the Pumani device connected to F&P nasal masks also generated appropriate levels of PP, except at very low liter flow rates (Fig. 9), as did utilizing a low-flow oxygen source connected to the Pumani tubing and prongs (Fig. 6). Interestingly, the combination of low flow oxygen source, F&P bubble chamber and Pumani tubing/interface differed from the complete Pumani data at high flow rates (Fig. 4). The differences observed between Fig. 4 and Fig. 6 and the small standard deviation measurements could be due to unmeasured leakage or differences in the actual air flow delivered by the Pumani air pump versus a low flow wall oxygen source.

F&P nasal prongs are designed to “fill the nares completely without stretching the skin” (F&P bCPAP brochure). This is to create a seal without causing tissue pressure injuries. In our testing, PP was only consistently achieved when there was 100% occlusion of the distal end of the apparatus (prongs or mask). If the apparatus was not occluded (as would be the case in older children), the PP delivered was necessarily less than the submersion depth. This observation has particular relevance when considering the implications of attempting to provide PP to older children in clinical contexts such as septic shock. When treating septic shock, PP (non-invasive or invasive) is often administered with a goal to not only reduce work of breathing, but also to reduce left ventricular afterload (assuming adequate preload), thereby reducing the overall metabolic consumption of oxygen which can help to optimize oxygen delivery [17]. In shock states, failure to achieve these goals can have detrimental, potentially fatal, effects. This physiology underscores the risk of using improvised low-tech bCPAP devices that are used outside of previously tested age-ranges.

Either soft cloth or gel-plastic nasal masks are the patient interface typically used to deliver CPAP or BIPAP to older children in resource-replete settings. Testing the Pumani device with a typical commercially available CPAP/BIPAP mask designed for older children (SleepWeaver™, Circadiance, Export, PA) did not generate any measurable PP (Table 1, data not shown) due to the inherent controlled leakage of the mask, which the low liter bias flow rate of Pumani was not able to overcome. Typical commercially available CPAP/BIPAP masks designed for older children have vented interfaces and require high liter bias flow rates of > 40 lpm to function appropriately and prevent CO_2_ rebreathing [18]. Importantly, in a RLS, flow rates greater than 10 lpm are practically hard to generate. Oxygen cylinders deplete quickly if used at > 10 lpm, raising stability of supply chain issues if the liter flow rate is raised > 10 lpm. Typical electric oxygen concentrators only generate < 10 lpm. Therefore, it may not be possible to provide CPAP/BIPAP to older children whose faces/noses are too large to fit a non-vented nasal mask (e.g. an F&P device) in the absence of reliable, built-in wall-piped-high-flow oxygen or air (rare in a RLS) or without a device employing a self-contained flow pump.

There are several limitations to this in vitro study. The devices and improvisations were tested in static conditions, in the absence of any type of lung simulator and were not attached to any type of simulated pharynx, preventing the estimation of pharyngeal pressures and any step down in pressure transmission [19]. With static testing, the choice of interface should not significantly affect the generated pressure unless the outlet resistance is markedly higher. This phenomenon was only observed with the smallest F&P prong set similar to prior observations, [14] where the authors observed a large increase in resistance for the F&P 3020 prongs independent of flow rate. We did test the F&P device with fully occluded and fully-unoccluded conditions, but this does not appropriately simulate the effect of having the mouth open or closed. We only tested flows < 10 lpm and did not measure air flow leakage for any device. Reduction of leakage is crucial for PP generation [15].

Although there have been some attempts to create higher flow devices with Venturi valves that can service older patients (e.g. ECPAP), [20] we are hopeful that the emergence of high flow nasal cannula devices which have built-in flow pumps and which can generate flows up to 60 lpm without a high-pressure wall air source (e.g. F&P Airvo™ 2 system), as well as the general robustness of the Pumani model, point to the possible future development of new innovative devices that could safely, successfully and reliably generate non-invasive positive pressure without high-pressure wall-piped air/oxygen in a RLS for older children.

## Conclusion

In well-resourced settings, a trial of non-invasive respiratory support to avoid endotracheal intubation and mechanical ventilation is generally accepted as safe and effective therapy for infants and children with moderate to severe respiratory illnesses. All of these interventions require high-level technical knowledge and robust supply chains which may be scarce in RLS [21]. Bubble CPAP has been proposed as an alternative in resource-limited settings to meet the need for respiratory support for infants [6]. However, there is an urgent need to develop reliable non-invasive positive pressure capacity for children above the infant age range.

All of the WHO-style bCPAP devices that we tested were able to generate PP that matched, or markedly exceeded, the submersion depth set in the associated bubble-chamber. PP was only measurable with 100% seal of the distal end of the bCPAP patient interfaces (commercial or improvised) but perfect seal is anatomically unlikely in older children. Therefore, the use of WHO-style bCPAP devices in patients outside the infant age range may be limited. Our data do support the effectiveness of bCPAP to generate appropriate pressures in newborns and young infants. However, based on our results, we recommend against using WHO-style bCPAP devices for non-infant patients with respiratory failure due to the low likelihood of any PP generation and instead recommend using standard oxygen therapy with nasal cannulae or face-masks, as well as early consideration of transfer to a higher level of care.

## Data Availability

All data generated or analyzed during this study are included in this published article as graphs and all raw data are available from the corresponding author on reasonable request.

## Abbreviations

RLS: Resource limited setting
CPAP: Continuous positive airway pressure
bCPAP: Bubble CPAP
lpm: Liters per minute
F&P: Fisher & Paykel
BIPAP: Bilevel positive airway pressure
PP: Positive pressure
